# The impact of a hospital-based exercise oncology program on cancer treatment–related side effects among rural cancer survivors

**DOI:** 10.1007/s00520-021-06010-5

**Published:** 2021-01-27

**Authors:** Adriana M. Coletta, Nathan B. Rose, Austin F. Johnson, D. Scott Moxon, Stephen K. Trapp, Darren Walker, Shelley White, Cornelia M. Ulrich, Neeraj Agarwal, Sonal Oza, Rebecca W. Zingg, Pamela A. Hansen

**Affiliations:** 1 Department of Health & Kinesiology, The University of Utah, Salt Lake City, UT USA; 2 The Huntsman Cancer Institute at the University of Utah, 2000 Circle of Hope Drive, Rm 4747, Salt Lake City, UT 84112 USA; 3 George E Wahlen, Department of Veterans Affairs Medical Center, Salt Lake City, UT USA; 4 Department of Population Health Sciences, The University of Utah, Salt Lake City, UT USA; 5 Department of Internal Medicine, The University of Utah, Salt Lake City, UT USA; 6 Division of Physical Medicine and Rehabilitation, The University of Utah, Salt Lake City, UT USA

**Keywords:** Exercise oncology, Rural cancer survivors

## Abstract

**Purpose:**

To assess the impact of the Personal Optimism With Exercise Recovery (POWER) program on cancer treatment–related side effects among rural cancer survivors.

**Methods:**

In this retrospective study of data collected between 2016 and 2019, we assessed change in cardiorespiratory fitness, whole-body muscular endurance, physical function and strength, anthropometrics, fatigue, and quality of life (QoL), after participation in POWER. Descriptive statistics were calculated for demographic and clinical variables. Univariate analysis of variance was carried out with age and BMI at initial assessment as covariates.

**Results:**

A total of 239 survivors, 78% rural residents, completed a follow-up assessment. Among rural cancer survivors, the most prevalent cancer sites were breast (42.5%), prostate (12.4%), and lymphoma (5.9%). The majority of survivors were female (70%), non-Hispanic (94.6%), and white (93.5%), with average age and BMI of 62.1 ± 13.2 years and 28.4 ± 6.7 kg/m^2^, respectively. Rural cancer survivors with cancer stages I–III exhibited significant improvements in fitness (+ 3.07 ml/kg/min, 95% CI 1.93, 4.21; + 0.88 METS, 95% CI 0.55, 1.20), physical function (30-s chair stand: + 2.2 repetitions, 95% CI 1.3, 3.1), muscular endurance (10-repetition maximum: chest press + 4.1 kg, 95% CI 2.0, 6.3; lateral pulldown + 6.6 kg, 95% CI 4.4, 8.9), self-reported fatigue (FACIT-Fatigue score: + 4.9, 95% CI 1.6, 8.1), and QoL (FACT-G7 score + 2.1, 95% CI, 0.9, 3.4). Among stage IV rural and urban cancer survivors, significant improvements were observed in muscular endurance and physical function.

**Conclusion:**

Participation in POWER was associated with attenuation of cancer treatment–related side effects and may serve as a model exercise oncology program for rural cancer survivors.

## Introduction

Nearly 20% of cancer survivors in the USA reside in rural areas [1]. Mortality rates of cancer survivors in rural areas are 9.6% greater compared with survivors in urban areas [1]. There is substantial evidence to support exercise across the cancer care continuum (e.g., before, during, and after cancer treatment) as an effective strategy to attenuate treatment-related side effects and improve health-related quality of life (QoL) [2–5]. Examples of side effects include declines in cardiorespiratory fitness, physical function, strength, and muscular endurance. Notably, declines in cardiorespiratory fitness are linked with increased mortality among cancer survivors [6–8]. Nationwide, evidence consistently demonstrates lower engagement in leisure time physical activity, or structured exercise, among cancer survivors in rural areas compared with urban areas [9, 10]. Access to exercise programs and education related to the importance of exercise for cancer survivorship are postulated as the primary reasons for this disparity [1, 11]. While evidence from randomized controlled trials support the feasibility, acceptability, and efficacy of exercise among rural cancer survivors [12, 13], there is a paucity of data related to the effectiveness of exercise oncology programs for rural cancer survivors.

The Huntsman Cancer Institute at the University of Utah (HCI), located in Salt Lake City, Utah, includes a catchment area that covers 17% of the continental US land mass, spanning across five, primarily rural, states: Utah, Wyoming, Montana, Idaho, and Nevada. As such, there are health promotion programs in place for cancer survivors that are unique to the rural nature of the catchment area. These programs aim to diminish known health disparities among individuals living in rural areas, such as lower rates of cancer screening and engagement in exercise across the cancer care continuum [1].

The Personal Optimism With Exercise Recovery (POWER) program is HCI’s long-standing (established in 2005) exercise oncology program aimed at facilitating exercise engagement across the cancer care continuum, mitigating cancer treatment–related side effects, and improving health-related QoL. POWER serves all survivors who seek care at HCI. POWER, like most exercise oncology programs offered through cancer institutions worldwide, provides personalized exercise programming, including aerobic and resistance exercise [4, 5, 14, 15]. Unique to POWER, options for program delivery are dictated by survivor preference. The methods available for program delivery include a supervised, home-based program delivered through the institution’s telemedicine platform; a supervised, in-person program; an unsupervised, home-based program; or a hybrid of the modes of program delivery. Considering the prevalence and mortality of cancer survivors in rural areas, the large, primarily rural, catchment area of HCI, and utility of exercise across the cancer continuum, the objective of this work is to assess the impact of HCI’s exercise oncology program, POWER, on treatment-related side effects and health-related QoL. We hypothesize that participation in the POWER program will associate with significant improvements in treatment-related side effects and health-related QoL among rural cancer survivors.

## Methods

### Study design and cancer survivor population

This is a retrospective analysis of data collected between 2016 and 2019 from HCI’s structured exercise oncology program, POWER. The protocol and waiver of informed consent was approved by the University of Utah Institutional Review Board. POWER is offered to all patients at the Huntsman Cancer Institute’s onsite Wellness and Integrative Health Center, and this includes patients with invasive cancer and those at high risk of developing cancer (for example, history of non-invasive cancer, such as ductal carcinoma in situ, or genetic predisposition, such as *BRCA1/2* mutation). Patients who participate in the POWER program are enrolled by provider or self-referral. While enrollment in the program can occur at any time across the cancer care continuum, most survivors who enroll are currently undergoing active treatment. The present investigation includes patients with a history of invasive cancer.

### Survivor demographic and clinical characteristics

Sex, race, and ethnicity were extracted from the medical record. The following data relate to the time the survivor began participating in the POWER program and was extracted from the medical record: cancer site, cancer stage, age, and body mass index (BMI). Variables related to cardiorespiratory fitness, whole-body muscular endurance, physical function and strength, anthropometrics, self-reported fatigue, and QoL were collected from the POWER program assessments.

Classification of geographic location (e.g., urban vs rural) was derived from zip code data included in the medical record. Population density maps for each state [16], available from the United States Department of Agriculture Economic Research Service (USDA-ERS), were cross-checked with both population estimates from the United States Census Bureau [17] and the USDA-ERS rural-urban continuum codes (RUCC) [18]. Urban geographical location was defined as approximately > 40,000 individuals residing in a specific zip code. Accordingly, rural was defined as a population approximately < 40,000 individuals. This is consistent with previous research that utilized RUCC 1–3 as urban location and RUCC ≥ 4 as rural [1, 9, 10, 19, 20].

### POWER program

POWER is based out of HCI’s onsite Wellness and Integrative Health Center. HCI’s Wellness and Integrative Health Center contains dedicated space for the POWER program gym, including aerobic exercise equipment (e.g., treadmills, bikes), free weights, weight machines, and resistance bands. The Wellness and Integrative Health Center also contains dedicated office space for providers and staff to document each session related to the POWER program in the medical record. In addition to the onsite clinic, the POWER program offers services at HCI’s community health centers.

POWER provides personalized exercise prescription that includes aerobic, resistance, balance, and flexibility training based on an in-person physical and medical assessment conducted in the POWER program gym by a team of physiatrists and certified exercise physiologists. The physical assessment includes the collection of vitals, anthropometrics (height, weight, waist and hip circumference), testing for cardiorespiratory fitness, whole-body muscular endurance, physical function and strength, and mobility and balance. Specifically, examples of testing include modified Bruce treadmill test, 6-min walk test, 30-s chair-stand, timed up and go, planks, single leg stand, back scratch, hip and shoulder abduction, flexion and extension, and ten-repetition maximum testing with leg press, chest press, and lateral pulldown. Fatigue and QoL are additionally assessed via Functional Assessment of Chronic Illness Therapy-Fatigue (FACIT-Fatigue) and Functional Assessment of Cancer Therapy-General (FACT-G7) questionnaires respectively. The medical assessment includes a review of the survivor’s past medical history and current cancer treatment regimen. The clinical exercise physiologists use information gathered from these assessments to design a personalized aerobic and whole-body resistance exercise prescription. Both the aerobic and resistance training components of each patient’s individualized exercise prescription is developed utilizing the FITT (frequency, intensity, time, type) principle and following the American College of Sports Medicine guidelines for exercise prescription in cancer survivors [2, 21]. It is important to note that if severe functional deficits are discovered during the assessment, the physiatrist will refer the survivor to the appropriate therapy service (e.g., physical therapy, occupational therapy). Participation in POWER may then occur in tandem or concurrently with the referred therapy service depending on the survivor’s needs.

The aerobic exercise prescription is developed with the goal to progress to or maintain aerobic exercise levels that meet the national physical activity guidelines (e.g., at least 150 min per week of moderate-intensity aerobic exercise, 75-min per week of vigorous-intensity aerobic exercise, or a combination of the two). The resistance exercise prescription is also developed to align with national physical activity guidelines (e.g., resistance training at least 2 days per week) and consists of up to 12 resistance exercises total, focusing on all major muscle groups including upper and lower body, core, and whole body. The resistance training prescription primarily consists of weight training (using equipment available or body weight only if indicated) and use of elastic resistance bands. Any side effects reported during exercise training resulted in modification of the workout and/or exercise program depending on the case.

Delivery of the aerobic and resistance training prescription is based on survivor preference and can be supervised in-person at the Wellness Center gym, home-based, or hybrid of the two. In the home-based setting, the aerobic and resistance training prescriptions may either be supervised or unsupervised, pending survivor preference and where appropriately recommended [21]. The majority of resistance training prescriptions include at least 1 day of supervised training per week. Supervised, home-based training is delivered through the University of Utah’s established telemedicine platform, EPIC Systems Corporation© (Verona, WI) MyChart® application. The telemedicine platform consists of HIPAA-compliant software that is run through EPIC electronic medical record system. Upon completion of the initial assessment, survivors who choose to complete their resistance training prescription at home receive an orientation regarding how to set up and use the telemedicine platform. Survivors can use the platform from any device that has a camera and can connect to the internet.

For survivors who are completing home-based programs, if they prefer free weights or resistance machines and have the equipment at home, their resistance training prescription may include this equipment. Alternatively, if a survivor prefers to use this equipment and complete training in-person at the Wellness Center’s gym, then the program will include this equipment. Otherwise, body weight training and elastic resistance bands are used as the mode for resistance training.

### Cardiorespiratory fitness

Cardiorespiratory fitness variables, relative peak aerobic capacity (ml/kg/min) and peak metabolic equivalents (METs), were estimated using a modified Bruce protocol and the American College of Sports Medicine’s metabolic calculations for the estimation of energy expenditure [21]. All tests were conducted on a treadmill and included a cooldown. Heart rate, oxygen saturation, and rating of perceived exertion, as assessed by the modified Borg scale (1–10), were recorded at rest, at the end of the warm-up and every stage during the exercise test, at peak aerobic capacity, and every minute during the cooldown.

### Whole-body muscular endurance

Ten-repetition maximum testing was carried out for chest press, lateral pulldown, and leg press in order to assess whole-body muscular endurance. Testing was carried out on a Paramount MP Series 4 Multi-Station Machine (Tartan Group, Burr Ridge, IL) following standard procedures [21].

### Physical function and strength testing

The timed up and go test, 30-s chair stand test, and right and left handgrip strength test were used to assess physical function and strength.

#### Timed up and go

Patients were instructed to get up from an arm chair, walk 3 m, turn around, walk back to the chair, and sit down [22, 23]. Patients were timed while completing this task. Lower times represent greater function and mobility.

#### Thirty-second chair stand

Patients began the test seated in a chair and were asked to stand up and sit down as many times as they could for 30 s [24]. Higher repetitions represent greater physical function.

#### Handgrip strength test

A portable, hydraulic dynamometer (Jamar 5030J1) was used to measure right and left handgrip strength, in kilograms, following a static handgrip strength protocol [25]. While seated, the patient was instructed to squeeze the instrument as hard as they could for 5 s. A total of three sets with a 2-min rest between sets was completed for each hand, and the best score was recorded. Higher scores indicate greater strength.

### Anthropometric assessment

Height and weight were measured following standard procedures. Waist circumference was measured about 2 in. above the umbilicus at the natural waistline. Hip circumference was measured at the widest point of the hips.

### Fatigue assessment

Fatigue was assessed by FACIT-Fatigue version 4 questionnaire. This instrument consists of 13 items and the reliability coefficient is about 0.90 [26]. Higher scores indicate lower fatigue.

### Quality of life assessment

QoL was assessed by the rapid version of the FACT-G7 questionnaire. This instrument consists of seven items related to symptoms and concerns that cancer patients view as the most important in terms of QoL [27]. The reliability coefficient is about 0.74 [27]. Higher scores indicate higher rating of QoL.

### Statistical methods

Descriptive statistics were calculated for demographic and clinical variables. Demographic and clinical variables assessed at the initial POWER assessment are presented as either frequency and percentages or mean and standard deviations. Mean change variables were computed as the difference between the follow-up assessment value and the initial assessment value. Univariate analysis of variance with age and BMI at initial assessment as covariates was carried out for the change from initial assessment to follow-up for variables collected from the POWER assessments. Assessment data are presented as means and 95% confidence intervals (CI). All data were analyzed with SPSS statistical software package, version 26 (Chicago, IL).

## Results

### Cancer survivor characteristics

A total of 849 survivors completed an initial assessment and participated in the POWER program. Table 1 outlines demographic and clinical characteristics of these individuals. The majority of survivors, 75%, lived in a rural setting. The most prevalent cancer sites were breast (34%), prostate (13%), and multiple myeloma (8%). The vast majority of survivors were female (62%), non-Hispanic (93%), and white (91%). Average age and BMI were 61.3 ± 13.6 years and 28.6 ± 6.7 kg/m^2^, respectively.

**Table 1:** Characteristics of survivors who completed an initial assessment and participated in POWER (*n* = 849)

Variable	*n*	%
Demographic characteristics		
Sex		
Female	527	62
Male	322	38
Race		
White	773	91
African American	4	0
Asian	13	2
Native Hawaiian and Other Pacific Islander	3	0
American Indian or Alaskan Native	1	0
Other	55	6
Ethnicity		
Hispanic	42	5
Non-Hispanic	791	93
Unknown	16	2
Geographical location		
Rural	638	75
Urban	211	25
Clinical characteristics		
Cancer site		
Bladder	4	0
Brain	24	3
Breast	288	34
Cervical	4	0
Colorectal	34	4
Endometrial	30	4
Fallopian tube	3	0
Gastrointestinal	13	2
Kidney	13	2
Leukemia	39	5
Liver	3	0
Lung	31	4
Lymphoma	43	5
Melanoma	22	3
MGUS	4	0
Multiple myeloma	70	8
Oral	9	1
Other	23	3
Ovarian	27	3
Pancreas	18	2
Peritoneum	4	0
Polycythemia vera	3	0
Prostate	114	13
Sarcoma	6	1
Skin	4	0
Testis	7	1
Thymus	2	0
Thyroid	5	1
Vaginal	2	0
Cancer stage		
Not staged	44	5
I	204	24
II	182	21
III	157	18
IV	158	19
Unknown	104	12
Age (years)	849	61.3 ± 13.5
BMI (kg/m^2^)	839	28.6 ± 6.6
Waist circumference (cm)	824	98.1 ± 17.0
Peak VO_2_ (ml/kg/min)	549	29.4 ± 7.6
Peak METs	549	8.4 ± 2.1
Fatigue (FACIT-Fatigue Score)	625	29.4 ± 11.7
Quality of life (FACT-G7 Score)	638	15.6 ± 5.0

*BMI* body mass index, *Peak VO*_*2*_ peak oxygen consumption, *METs* metabolic equivalents, *FACIT* Functional Assessment of Chronic Illness Therapy, *FACT* Functional Assessment of Cancer Therapy

Among the 849 survivors who completed an initial assessment and participated in the program, 239 elected to complete the in-person follow-up assessment to assess progress. Average length of time between initial assessment and follow-up assessment was 25 weeks. Two survivors elected a hybrid of home-based and in-person exercise training; the remaining 237 elected for in-person training at the Wellness Center gym. Table 2 outlines demographic and clinical characteristics of survivors who completed a follow-up assessment by the total sample (*n* = 239) and by geographic location (urban vs. rural). About 78% of survivors who completed a follow-up assessment resided in rural areas and 22% resided in urban areas. Among survivors in rural areas, the most prevalent cancer sites were breast (42.5%), prostate (12.4%), and lymphoma (5.9%). The majority were female (70%), non-Hispanic (95%), and white (94%). Average age and BMI were 61.5 ± 13 years and 28.27 ± 6.44 kg/m^2^.

**Table 2:** Characteristics of survivors who completed a follow-up assessment (*n* = 239)

Demographic characteristics			
Variable	*n* (%)		
	Rural, *n* = 186	Urban, *n* = 53	Total, *n* = 239
Sex			
Female	130 (70)	39 (74)	169 (71)
Male	56 (30)	14 (26)	70 (29)
Race			
White	174 (93.5)	51 (96)	225 (94)
Asian	2 (1.1)	0	2 (0.8)
Other	10 (5.4)	2 (4)	12 (5)
Ethnicity			
Hispanic	8 (4.3)	3 (5.7)	11 (5)
Non-Hispanic	176 (94.6)	50 (94.3)	226 (95)
Unknown	2 (1.1)	0	2 (0.8)
Geographical location			
Rural	186 (100)	NA	186 (78)
Urban	NA	53 (100)	53 (22)
Clinical characteristics			
Variable	*n* (%)		
	Rural, *n* = 186	Urban, *n* = 53	Total, *n* = 239
Cancer site			
Bladder	1 (0.5)	0	1 (0.4)
Brain	3 (1.6)	1 (1.9)	4 (1.7)
Breast	79 (42.5)	16 (30.2)	95 (39.7)
Colorectal	4 (2.2)	3 (5.7)	7 (2.9)
Endometrial	9 (4.8)	6 (11.3)	15 (6.3)
Fallopian tube	1 (0.5)	0	1 (0.4)
Gastrointestinal	2 (1.1)	0	2 (0.8)
Kidney	3 (1.6)	0	3 (1.3)
Leukemia	11 (5.9)	2 (3.8)	10 (4.2)
Lung	3 (1.6)	3 (5.7)	6 (2.5)
Lymphoma	11 (5.9)	4 (7.5)	15 (6.3)
Melanoma	9 (4.8)	0	9 (3.8)
Multiple myeloma	6 (3.2)	4 (7.5)	10 (4.2)
Oral	1 (0.5)	1 (1.9)	2 (0.8)
Other	3 (1.6)	3 (5.7)	6 (2.5)
Ovarian	7 (3.8)	2 (3.8)	9 (3.8)
Pancreas	4 (2.2)	1 (1.9)	5 (2.1)
Peritoneum	3 (1.6)	0	3 (1.3)
Polycythemia vera	2 (1.1)	0	2 (0.8)
Prostate	23 (12.4)	3 (5.7)	26 (10.9)
Sarcoma	0	1 (1.9)	1 (0.4)
Skin	1 (0.5)	2 (3.8)	3 (1.3)
Testis	2 (1.1)	0	2 (0.8)
Thymus	1 (0.5)	1 (1.9)	2 (0.8)
Thyroid	7 (3.8)	2 (3.8)	9 (3.8)
Cancer stage			
Not staged	9 (4.6)	2 (3.8)	11 (4.6)
I	55 (27.9)	13 (24.5)	66 (27.6)
II	44 (22.3)	8 (15.1)	50 (20.9)
III	36 (18.3)	12 (22.6)	45 (18.8)
IV	28 (14.2)	9 (17)	35 (14.6)
Unknown	25 (12.7)	9 (17)	32 (13.4)
Variable	Mean ± SD (*n*)		
	Rural	Urban	Total
Age (years)	62.1 ± 13.2 (186)	59.6 ± 13.4 (53)	61.5 ± 13.2 (239)
BMI (kg/m^2^)	28.4 ± 6.7 (185)	27.9 ± 5.3 (52)	28.3 ± 6.4 (237)
Waist circumference (cm)	96.3 ± 17.3 (177)	93.1 ± 14.4 (52)	95.6 ± 16.7 (229)
Peak VO_2_ (ml/kg/min)	30.2 ± 7.8 (125)	32.0 ± 7.4 (39)	30.6 ± 7.7 (164)
Peak METs	8.6 ± 2.2 (125)	9.1 ± 2.1 (39)	8.7 ± 2.2 (164)
Fatigue (FACIT-Fatigue Score)	30.7 ± 12.2 (147)	29.8 ± 13.6 (44)	30.5 ± 12.5 (191)
Quality of life (FACT-G7 Score)	16.5 ± 5.1 (146)	15.7 ± 5.7 (47)	16.3 ± 5.2 (193)

*BMI* body mass index, *Peak VO*_*2*_ peak oxygen consumption, *METs* metabolic equivalents, *FACIT* Functional Assessment of Chronic Illness Therapy, *FACT* Functional Assessment of Cancer Therapy, *NA* not available

### Impact of the exercise oncology program

Among survivors who completed follow-up assessments, significant favorable changes at follow-up were observed for cardiorespiratory fitness, physical function, muscular endurance, and self-reported fatigue and QoL, but not for anthropometric measurements or handgrip strength. These findings were also observed after adjusting for age and BMI from the initial assessment: cardiorespiratory fitness: + 2.7 ml/kg/min (95% CI 1.9, 3.6), + 0.8 METS (95% CI 0.5, 1.0); physical function: timed up and go − 0.37 s (95% CI − 0.62, − 0.11), 30-s chair stand + 2 repetitions (95% CI 1.8, 3.1); muscular endurance (10 RM): chest press + 5.2 kg (95% CI 3.8, 6.7), lateral pulldown + 7.2 kg (95% CI 5.6, 8.8), leg press + 31.4 kg (95% CI 5.9, 57.0); self-reported fatigue: FACIT-fatigue score + 3.7 (95% CI 1.6, 6.0); QoL: FACT-G7 score + 1.6 (95% CI, 0.7, 2.4); waist circumference + 0.34 cm (95% CI − 0.36, 1.03), hip circumference − 0.12 cm (95% CI − 0.66, 0.41); right handgrip strength + 0.41 kg (95% CI − 0.47, 1.29), and left handgrip strength + 0.08 kg (95% CI − 0.77, 0.93).

Table 3 outlines the mean change in physical outcomes after participation in the POWER program by geographical location and cancer stage, adjusted for age and BMI. Regardless of geographical location, survivors with a cancer diagnosis of stage I, II, or III experienced significant improvements in cardiorespiratory fitness, muscular endurance, and physical function variables. Survivors who resided in rural areas also experienced significant improvements in fatigue and QoL; however, improvements in these variables were not observed among survivors who resided in urban areas. Among survivors with advanced cancer, regardless of geographical location, significant improvements were observed for variables of muscular endurance and physical function.

**Table 3:** Mean change in physical outcomes after participation in the POWER program

Variable	Urban			Rural		
	*n*	Mean	95% CI	*n*	Mean	95% CI
Cancer stages I–III						
Peak VO_2_ (ml/kg/min)	20	3.08	(1.27, 4.89)*	80	3.07	(1.93, 4.21)*
Peak METs	20	0.88	(0.36, 1.40)*	80	0.88	(0.55, 1.20)*
10 RM chest press (kg)	24	6.9	(3.1, 10.6)*	82	4.1	(2.0, 6.3)*
10 RM leg press (kg)	18	24.4	(10.7, 38.2)*	75	37.1	(− 10.0, 84.1)
10 RM Lat pulldown (kg)	23	8.3	(3.7, 12.8)*	83	6.6	(4.4, 8.9)*
Timed up and go (s)	32	− 0.45	(− 0.73, − 0.16)*	117	− 0.15	(− 0.56, 0.25)
30-s chair stand (repetitions)	30	2.8	(0.5, 5.2)*	123	2.2	(1.3, 3.1)*
Right hand grip (kg)	32	0.3	(− 2.5, 3.0)	126	0.6	(− 0.3, 1.5)
Left hand grip (kg)	32	− 0.2	(− 2.7, 2.2)	126	0.7	(− 0.2, 1.7)
Waist circumference (cm)	30	0.47	(− 1.27, 2.20)	122	0.20	(− 0.72, 1.12)
Hip circumference (cm)	30	− 0.98	(− 2.63, 0.66)	123	−0.13	(− 0.85, 0.59)
FACIT-Fatigue Score	21	2.3	(− 5.7, 10.2)	84	4.9	(1.6, 8.1)*
FACT-G7 Score	23	1.7	(− 0.7, 4.2)	84	2.1	(0.9, 3.4)*
Cancer stage IV						
Peak VO_2_ (ml/kg/min)	7	3.54	(− 1.16, 8.24)	13	1.50	(− 2.47, 5.46)
Peak METs	7	1.01	(− 0.33, 2.36)	13	0.43	(− 0.55. 1.5)
10 RM chest press (kg)	6	6.7	(− 0.8, 14.1)	16	7.3	(3.6, 11.0)*
10 RM leg press (kg)	5	53.5	(26.5, 80.6)*	15	17.0	(6.4, 27.6)*
10 RM Lat pulldown (kg)	7	6.4	(2.5, 10.4)*	16	9.1	(4.5, 13.6)*
Timed up and go (s)	7	− 0.2	(− 0.4, 0.01)	22	− 1.1	(− 2.0, − 0.14)*
30-s chair stand (repetitions)	7	3.0	(0.3, 5.8)*	24	2.3	(1.0, 3.5)*
Right hand grip (kg)	8	1.4	(− 3.1, 5.8)	24	− 2.0	(− 7.9, 4.0)
Left hand grip (kg)	8	0.3	(− 3.5, 4.0)	24	− 2.9	(− 8.1, 2.4)
Waist circumference (cm)	7	0.79	(− 2.63, 4.20)	22	0.18	(− 3.01, 3.38)
Hip circumference (cm)	8	0.63	(− 1.65, 2.90)	22	− 0.98	(− 2.62, 0.67)
FACIT-Fatigue Score	7	2.4	(− 16.3, 21.1)	17	2.9	(− 1.6, 7.4)
FACT-G7 Score	7	0.7	(− 7.7, 9.1)	16	− 0.4	(− 2.5, 1.8)

*Peak VO*_*2*_ peak oxygen consumption, *METs* metabolic equivalents, *10 RM* 10-repetition maximum, *Lat* lateral, *FACIT* Functional Assessment of Chronic Illness Therapy, *FACT* Functional Assessment of Cancer Therapy

*Statistically significant (*p* < 0.05). Results adjusted for age and BMI at initial assessment

## Discussion

We aimed to assess the impact of our exercise oncology program, POWER, on physical cancer treatment–related side effects and health-related QoL among cancer survivors who electively participated in our program and completed a follow-up assessment to evaluate progress. We found that participation in POWER was associated with favorable changes in side effects and QoL. These findings were observed among varying cancer types, regardless of cancer stage and geographical location, supporting the feasibility, effectiveness, and generalizability of the POWER exercise oncology program.

There are several exercise oncology programs worldwide offered by cancer hospitals and community organizations that have published the impact of their program on exercise engagement, physical outcomes, and QoL. Examples of these programs include the ActivOnco Model in Montreal, Quebec [14], the Community-Level Cancer Rehabilitation Program in Copenhagen, Denmark [15], The Wellness and Exercise for Cancer Survivors Program in Ontario, Canada [5], the Livestrong at the YMCA program offered in communities across the USA [28], and the Strides to Strength Cancer Rehabilitation Program in Charlotte, North Carolina [4]. Our findings add to the current body of literature such that participation in exercise oncology programs is associated with promoting exercise engagement, attenuating cancer treatment–related side effect, and improving QoL [4, 5, 15, 28–31]. Consistent with some of these programs, POWER is coordinated by a cancer center [4, 5, 14]. This is important as exercise programs coordinated by cancer centers facilitate exercise engagement among cancer survivors [5, 32, 33]. Additionally, POWER is geared towards graduated development of patient independence in a home-based program, whether this commences with in-person gym sessions or telemedicine. It is recognized that home-based exercise is a prevailing facilitator to exercise engagement among cancer survivors [32, 34].

POWER’s advantage is its fairly large, primarily rural, catchment area and provision of choice for program delivery. Markedly, POWER, to our knowledge, is the first exercise oncology program in the USA to offer home-based exercise training delivered through the cancer institution’s electronic medical record system. The pre-existence of our telemedicine option allowed for a seamless transition to telemedicine during the COIVD-19 pandemic. Additionally, the supervision contributes to external accountability, a known facilitator to exercise engagement specifically among rural cancer survivors [33]. Furthermore, POWER utilizes expertise from rehabilitation professionals, notably physiatrists, and clinical exercise physiologists in order to effectively deliver individualized exercise prescription to cancer survivors of all types and stages. The integration of expertise from these professions is critical for exercise programming, as this indicates consideration of patient-specific neuro-musculoskeletal concerns, functional deficits, and physical deconditioning [35].

It is important to note that other exercise oncology programs nationwide do not, to our knowledge, have published data on outcomes. The American College of Sports Medicine has recently developed a searchable registry within their *Moving Through Cancer* initiative, where individuals can find exercise oncology programs in their communities [36]. The registry is organized by country (and in the USA by state) and program type (e.g., inpatient/outpatient hospital, community, home-based, research study) to best meet the preferences of the cancer survivor [36]. Thirty-five of the 50 states in the USA include at least one program, yet, we understand only two have published data on outcomes [4, 29]. Considering our ability to reach cancer survivors in rural areas, while also synergizing expertise from rehabilitation and exercise professionals to optimize exercise program delivery, we believe POWER may serve as a model exercise oncology program for cancer centers nationwide. We consider these components strengths of our program.

Along with our strengths, our work is not without its limitations. The presented data is heterogeneous in nature, making it difficult to discern benefits by cancer type due to smaller sample sizes within each cancer type. Nearly one-third of survivors who elected to participate in the program completed a follow-up assessment to track progress. In addition, not all survivors were able to complete all tests at their follow-up assessments due to limitations from treatment, leaving varying sample sizes for each variable at follow-up. Future work should assess more data over a longer period of time in order to increase sample sizes in each cancer type. Exploration of possible reasons why survivors elected not to complete a follow-up assessment would be helpful in understanding how to improve design of evaluating progress in the exercise oncology program. Furthermore, future work should assess cost-effectiveness and cost-savings of this program over time in order to demonstrate to third-party payers the utility of reimbursing exercise services across the cancer care continuum.

## Conclusions

Overall, the POWER program at the Huntsman Cancer Institute at the University of Utah is a long-standing, patient-centered exercise oncology program that addresses all of the significant facilitators to exercise engagement among cancer survivors, and is associated with both attenuating cancer treatment–related side effects and improving health-related quality of life among rural and urban cancer survivors. Findings from this study support hospital-based exercise oncology programs’ utility and the need for programs like POWER nationwide as part of the standard of cancer care to improve outcomes linked with survival and quality of life for cancer survivors.

## Data Availability

Data is available upon request.

## References

[1] Blake KD, Moss JL, Gaysynsky A, Srinivasan S, Croyle RT (2017). Making the case for investment in rural cancer control: an analysis of rural cancer incidence, mortality, and funding trends. Cancer Epidemiol Biomark Prev.

[2] Campbell KL, Winters-Stone KM, Wiskemann J, May AM, Schwartz AL, Courneya KS, Zucker DS, Matthews CE, Ligibel JA, Gerber LH, Morris GS, Patel AV, Hue TF, Perna FM, Schmitz KH (2019). Exercise guidelines for cancer survivors: consensus statement from international multidisciplinary roundtable. Med Sci Sports Exerc.

[3] Stout NL, Baima J, Swisher AK, Winters-Stone KM, Welsh J (2017). A systematic review of exercise systematic reviews in the cancer literature (2005-2017). PM R.

[4] Kirkham AA, Klika RJ, Ballard T, Downey P, Campbell KL (2016). Effective translation of research to practice: hospital-based rehabilitation program improves health-related physical fitness and quality of life of cancer survivors. J Natl Compr Cancer Netw.

[5] Santa Mina D, Au D, Auger LE, Alibhai SMH, Matthew AG, Sabiston CM, Oh P, Ritvo PG, Chang EB, Jones JM (2019). Development, implementation, and effects of a cancer center’s exercise-oncology program. Cancer.

[6] Vainshelboim B, Lima RM, Myers J (2019). Cardiorespiratory fitness and cancer in women: a prospective pilot study. J Sport Health Sci.

[7] Vainshelboim B, Müller J, Lima RM, Nead KT, Chester C, Chan K, Kokkinos P, Myers J (2017). Cardiorespiratory fitness, physical activity and cancer mortality in men. Prev Med.

[8] Lakoski SG, Willis BL, Barlow CE, Leonard D, Gao A, Radford NB, Farrell SW, Douglas PS, Berry JD, DeFina LF, Jones LW (2015). Midlife cardiorespiratory fitness, incident cancer, and survival after cancer in men: the Cooper Center Longitudinal Study. JAMA Oncol.

[9] Mama SK, Bhuiyan N, Foo W, Segel JE, Bluethmann SM, Winkels RM, Wiskemann J, Calo WA, Lengerich EJ, Schmitz KH (2020). Rural-urban differences in meeting physical activity recommendations and health status in cancer survivors in central Pennsylvania. Support Care Cancer.

[10] Weaver KE, Palmer N, Lu L, Case LD, Geiger AM (2013). Rural-urban differences in health behaviors and implications for health status among US cancer survivors. Cancer Causes Control.

[11] Bolin JN, Bellamy GR, Ferdinand AO, Vuong AM, Kash BA, Schulze A, Helduser JW (2015). Rural Healthy People 2020: new decade, same challenges. The Journal of rural health : official journal of the American Rural Health Association and the National Rural Health Care Association.

[12] Gray MS, Judd SE, Sloane R, Snyder DC, Miller PE, Demark-Wahnefried W (2019). Rural-urban differences in health behaviors and outcomes among older, overweight, long-term cancer survivors in the RENEW randomized control trial. Cancer Causes Control.

[13] Frensham LJ, Parfitt G, Dollman J (2018) Effect of a 12-week online walking intervention on health and quality of life in cancer survivors: a quasi-randomized controlled trial. Int J Environ Res Public Health 15(10). 10.3390/ijerph15102081 10.3390/ijerph15102081 PMC6210292 30248943

[14] Dalzell M, Smirnow N, Sateren W, Sintharaphone A, Ibrahim M, Mastroianni L, Vales Zambrano L, O'Brien S (2017). Rehabilitation and exercise oncology program: translating research into a model of care. Curr Oncol.

[15] Kristiansen M, Adamsen L, Pill K, Halvorsen I, Nyholm N, Hendriksen C (2017) A three-year national follow-up study on the development of community-level cancer rehabilitation in Denmark. Scand J Public Health:1–8 10.1177/1403494817746535 29212431

[16] USDA Economic Research Service: Rural Definitions. https://ers.usda.gov/data-products/rural-definitions/

[17] United State Census Bureau. Population Estimate (as of July 2018). https://factfinder.census.gov/faces/nav/jsf/pages/community_facts.xhtml

[18] United States Department of Agriculture (USDA). Rural-urban continuum codes: USDA economic research service [updated October 25, 2019]. https://www.ers.usda.gov/data-products/rural-urban-continuum-codes/

[19] Fan JX, Wen M, Kowaleski-Jones L (2014). Rural-urban differences in objective and subjective measures of physical activity: findings from the National Health and Nutrition Examination Survey (NHANES) 2003-2006. Prev Chronic Dis.

[20] Robertson MC, Song J, Taylor WC, Durand CP, Basen-Engquist KM (2018). Urban-rural differences in aerobic physical activity, muscle strengthening exercise, and screen-time sedentary behavior. The Journal of Rural Health.

[21] Riebe D, Ehrman JK, Ligouri G, Magai M, American College of Sports Medicine (2018). ACSM’s guidelines for exercise testing and prescription.

[22] Nicolini-Panisson RD, Donadio MV (2013). Timed “Up & Go” test in children and adolescents. Rev Paul Pediatr.

[23] Podsiadlo D, Richardson S (1991). The timed “Up & Go”: a test of basic functional mobility for frail elderly persons. J Am Geriatr Soc.

[24] Bohannon RW (1995). Sit-to-stand test for measuring performance of lower extremity muscles. Percept Mot Skills.

[25] Gerodimos V, Karatrantou K, Psychou D, Vasilopoulou T, Zafeiridis A (2017). Static and dynamic handgrip strength endurance: test-retest reproducibility. J Hand Surg Am.

[26] Yellen S, Cella D, Webster K, Blendowski C, Kaplan E (1997). Measuring fatigue and other anemia-related symptoms with the Functional Assessment of Cancer Therapy (FACT) measurement system. J Pain Symptom Manag.

[27] Yanez B, Pearman T, Lis CG, Beaumont JL, Cella D (2013). The FACT-G7: a rapid version of the functional assessment of cancer therapy-general (FACT-G) for monitoring symptoms and concerns in oncology practice and research. Ann Oncol.

[28] Heston A, Schwartz A, Justice-Gardiner H, Hohman K (2015). Addressing physical activity needs of survivors by developing a community-based exercise program: LIVESTRONG® at the YMCA. Clin J Oncol Nurs.

[29] Irwin ML, Cartmel B, Harrigan M, Li F, Sanft T, Shockro L, O'Connor K, Campbell N, Tolaney SM, Mayer EL, Yung R, Freedman RA, Partridge AH, Ligibel JA (2017). Effect of the LIVESTRONG at the YMCA exercise program on physical activity, fitness, quality of life, and fatigue in cancer survivors. Cancer.

[30] Rossen S, Trier K, Christensen B, Eriksen MA, Zwisler AD, Vibe-Petersen J (2020). Municipality-based pragmatic rehabilitation stratified in accordance with individual needs-results from a longitudinal survey study. Support Care Cancer.

[31] Schumacher MM, McNiel P (2018). The impact of Livestrong® at the YMCA for cancer survivors. Oncol Nurs Forum.

[32] Wong JN, McAuley E, Trinh L (2018). Physical activity programming and counseling preferences among cancer survivors: a systematic review. Int J Behav Nutr Phys Act.

[33] Hardcastle SJ, Galliott M, Lynch BM, Nguyen NH, Cohen PA, Mohan GR, Johansen NJ, Saunders C (2019). ‘If I had someone looking over my shoulder…’: exploration of advice received and factors influencing physical activity among non-metropolitan cancer survivors. Int J Behav Med.

[34] Hardcastle SJ, Maxwell-Smith C, Kamarova S, Lamb S, Millar L, Cohen PA (2018). Factors influencing non-participation in an exercise program and attitudes towards physical activity amongst cancer survivors. Support Care Cancer.

[35] Coletta AM, Campbell A, Morris GS, Schmitz KH (2020). Synergy between licensed rehabilitation professionals and clinical exercise physiologists: optimizing patient care for cancer rehabilitation. Semin Oncol Nurs.

[36] Exercise is Medicine. American College of Sports Medicine. Moving Through Cancer. https://www.exerciseismedicine.org/support_page.php/moving-through-cancer/

